# Squalene-Rich Amaranth Oil Pickering Emulsions Stabilized by Native α-Lactalbumin Nanoparticles

**DOI:** 10.3390/foods11141998

**Published:** 2022-07-06

**Authors:** Andrea P. Cuevas-Gómez, Berenice González-Magallanes, Izlia J. Arroyo-Maya, Gustavo F. Gutiérrez-López, Maribel Cornejo-Mazón, Humberto Hernández-Sánchez

**Affiliations:** 1 Departamento de Ingeniería Bioquímica, Escuela Nacional de Ciencias Biológicas, Instituto Politécnico Nacional, Mexico City C.P. 07738, Mexico; andreac327@gmail.com (A.P.C.-G.); bgm-1621@hotmail.com (B.G.-M.); gusfgl@gmail.com (G.F.G.-L.); 2 División de Ciencias Naturales e Ingeniería, Universidad Autónoma Metropolitana Cuajimalpa, Mexico City C.P. 05348, Mexico; 3 Departamento de Biofísica, Escuela Nacional de Ciencias Biológicas, Instituto Politécnico Nacional, Mexico City C.P. 11340, Mexico; maribelpabe2@hotmail.com

**Keywords:** amaranth oil, Pickering emulsion, α-lactalbumin, nanoparticles

## Abstract

The stabilization of Pickering emulsions by nanoparticles has drawn great interest in the field of food science and technology. In this study, α-Lactalbumin nanoparticles prepared by the desolvation and cross-linking method from protein solutions with initial pH values of 9 and 11 were used to stabilize squalene-rich amaranth oil Pickering o/w emulsions. The effect of different concentrations of nanoparticles on the size, size distribution, ζ potential, and emulsion stability was evaluated using dynamic light scattering, electron microscopy, and light backscattering. Dependence of the emulsions’ droplet size on the nanoparticle concentration was observed, and the critical coverage ratio was reached when 5–10% nanoparticles concentration was used. Our findings suggest that α-LA nanoparticles at a 10% concentration can be used as novel stabilizers for Pickering emulsions to provide protection for beneficial lipophilic bioactive compounds. This is the first time that native α-LA nanoparticles have been used as stabilizers of Pickering emulsions.

## 1. Introduction

Pickering emulsions are emulsions in which solid colloidal particles are used instead of molecular surfactants to convey kinetic stability [1]. Pickering emulsions are more stable than the “traditional” emulsions since the solid particles bind firmly to the oil-water interface, creating a stiff barrier against coalescence [2]. Natural macromolecules such as zein [3], soy [4] or whey proteins [5], and cellulose or starch particles [6] have been used as Pickering stabilizers for food applications due to their biocompatibility, biodegradability, and low cost [7,8]. Emulsions are adequate options for delivering different kinds of lipophilic bioactive compounds which are poorly soluble in water, chemically unstable, and sensitive to oxidation such as α-tocopherol [6], conjugated linoleic acid [7], polyphenols, and flavonoids [9]. Squalene was selected to be encapsulated in a Pickering emulsion since this compound has all the disadvantages mentioned above for hydrophobic compounds. The preparation of food-grade squalene-rich amaranth oil Pickering emulsions would provide a system containing a very valuable oil stabilized by edible solid protein particles which can be used for the formulation of functional foods (usually spread-like products) or dietary supplements.

Squalene is a natural linear triterpene, structurally similar to β-carotene, that can be found abundantly in shark liver oil, and to a lesser extent in a broad variety of vegetable oils such as amaranth, olive, and rice bran oils [10,11]. Squalene is well known for its key role as an intermediate in cholesterol synthesis [12]. Its bioactive properties have been widely studied. In vitro and in vivo studies revealed strong antioxidant properties which were able to reduce stress-induced intracellular reactive oxygen species (ROS). In the skin, squalene can act as an emollient and UV-protective compound. Additionally, squalene reduces pro-inflammatory cytokine secretion resulting in an overall protective role against uncontrolled inflammatory response [11]. There are also several studies indicating favorable effects of squalene-enriched diets on some cardiovascular pathologies. These effects included a decrease in plasma cholesterol and triglyceride levels, liver fat levels, and a reduction in systolic blood pressure [13].

Shark-derived squalene has been providing the global market needs since its initial discovery. Nevertheless, intensive fishing has had a damaging effect on oceanic ecosystems and endangered the populations of squalene-producing sharks [11]. The use of vegetable oils to obtain squalene has been considered then, as a more sustainable alternative. Amaranth grain contains 6 to 9% of oil which is higher than most cereal sources. Amaranth oil contains approximately 77% unsaturated fatty acids including linoleic acid. Additionally, the oil fraction is unique due to its high squalene content (2.4–8.0%). Since the clinical and nutraceutical applications of squalene are continuously growing, the demand for this substance will likely increase and amaranth oil could be a good alternative source [14,15]. One of the disadvantages of amaranth oil is its high susceptibility to oxidation, so the preparation of Pickering emulsions would be a good method to protect the oil and its valuable squalene content.

Previous studies have shown that whey protein isolate nanoparticles [5] and nanofibrils [7] can be used as stabilizers in Pickering emulsion systems to improve the stability of lipophilic bioactive compounds. In this study, the use of nanoparticles of one of the proteins (α-lactalbumin) from bovine whey is proposed as a stabilizer for Pickering emulsions containing squalene-rich amaranth oil. Bovine α-lactalbumin (α-LA) constitutes around 20 to 25% of the whey proteins, being the second most abundant protein in whey. It has a molecular weight of 14,178 Da, its isoelectric point is 4.2 to 4.6, it is very soluble in water and NaCl solutions, and it is relatively thermostable when bound to calcium [16]. It is the regulatory element in the lactose synthase complex, and it is involved in the modulation of the specificity of the galactosyl transferase subunit [17]. α-LA has been used previously to encapsulate lipophilic bioactive compounds such as retinol, α-tocopherol, and vitamin D_3_ [18,19]; however, the preparation of α-LA nanostructures as possible carriers for drug delivery and food applications has acquired great interest [20]. Protein nanostructures have been primarily used for the encapsulation and controlled release of bioactive compounds in food systems. They offer numerous advantages including a lack of toxicity, in vivo degradation, and aqueous solubility and stability as in the case of α-LA [20]. These nanostructures can be prepared by the self-assembly of α-LA hydrolysates or by different physical or chemical treatments on the native α-LA protein. The controlled hydrolysis of the α-LA can produce by spontaneous assembly nanotubes [21], nanodisks [22], and nanospheres [23], which can be utilized for the delivery of bioactive compounds [24]. Self-assembled α-LA-derived peptides have been used as Pickering stabilizers for oil-in-water emulsions of curcumin [23]. When the native α-LA is to be used as the raw material for nanoparticle formation, the methods of desolvation with ethanol or acetone followed by chemical cross-linking [25,26] or enzymatic crosslinking with microbial transglutaminase or horseradish peroxidase [27,28] have been used. However, the preparation of Pickering emulsions stabilized by native α-LA nanoparticles has not been explored to the better of our knowledge. The aim of this study was, then, to study the conditions to prepare squalene-rich amaranth oil Pickering emulsions stabilized by α-LA nanoparticles. To accomplish this, α-LA nanoparticles, prepared at pH 9 and 11 using a desolvation and cross-linking process, were added at different concentrations to stabilize amaranth oil Pickering emulsions developed by ultrasound and rotor-stator homogenization. The characteristics, stability, and microstructure of the oil-in-water Pickering emulsions were studied to define the preparation conditions for maximal stability.

## 2. Materials and Methods

### 2.1. Materials

α-LA (BiPRO^®^ Alpha 9000) was generously provided by Agropur Ingredients (Eden Prairie, MN, USA), amaranth oil was purchased from Gastronomía Molecular (Mexico City, Mexico), and all the other chemicals were bought from Sigma-Aldrich (St. Louis, MO, USA) and J.T. Baker (Mexico City, Mexico). They were of analytical grade and used as obtained.

### 2.2. Preparation of Native α-LA Nanoparticles

The α-LA nanoparticles were prepared according to the desolvation and cross-linking method described by Arroyo–Maya et al. [25]. Briefly, the α-LA (40 mg) was dissolved in 2 mL of a 10 mM NaCl solution (pH 9) or a 10 mM NaOH solution (pH 11) followed by the desolvation of the protein in solution by the controlled (1 mL/min) dropwise addition of 8 mL of acetone with constant mixing (500 rpm). After the desolvation step, 40 μL of 8% aqueous glutaraldehyde solution was added to attain the cross-linking of the α-LA. After a 3 h stirring step, the resultant nanoparticles were purified by five cycles of centrifugation (11500 rpm, 30 min, 4 °C). The final pellet was redispersed in the original volume of a 10 mM NaCl solution (pH 9.0) or a 10 mM NaOH solution (pH 11) using a Cole-Parmer 130-Watt Ultrasonic Processor. All preparations were performed at room temperature (25 °C).

### 2.3. Measurements of ζ Potential, Particle Size, and Size Distribution of the α-LA Nanoparticles

The ζ potential, particle size, and polydispersity index of the α-LA nanoparticles were measured immediately after preparation utilizing a Zetasizer Nano ZS90 (Malvern Instruments Inc., Malvern, U.K.) dynamic light scattering (DLS) instrument at 25 °C [26].

### 2.4. Microscopy Analysis of the α-LA Nanoparticles

The morphology of the α-LA nanoparticles was examined with a JEM1010 transmission electron microscope (JEOL, Tokyo, Japan) at 60 kV. The water suspension of the nanoparticles was diluted 10 times, and an aliquot of 5 μL was drop-casted onto a formvar carbon-coated copper grid (200 mesh); the grid was dried with air at room temperature before TEM imaging [25].

The morphometric characteristics of the nanoparticles were analyzed with a Quanta 200 FEG (FEI Company, Hillsboro, OR, USA) Dual Beam environmental scanning electron microscope using acceleration voltages of 10 and 20 kV for magnifications of 25,000 and 50,000 x respectively. The water suspension of the nanoparticles was diluted at 1:100 at 25 °C before observation [29].

The size and morphometric characteristics of the nanoparticles were also evaluated with a Nanosurf Naio Atomic Force Microscope (Liestal, Switzerland). The samples (10 μL) were allowed to dry at room temperature. The images were taken in dynamic force (tapping) mode with a Tap190Al-G probe [30]. All the observations were performed with freshly prepared nanoparticles.

### 2.5. Preparation of the Pickering Emulsions

Pickering o/w emulsions stabilized by the native α-LA nanoparticles obtained at pH 9 (NP1) and pH 11 (NP2) were prepared as described by Leal-Castañeda et al. [6] by mixing 17.5 mL of water with 7.5 mL of squalene oil and nanoparticles (NP1 and NP2) to reach concentrations of 0, 3, 5, 10, 15, and 20% wt. The mixtures were blended at 11,000 rpm for 3 min at 25 °C with an IKA T18 Basic Ultra-Turrax rotor-stator homogenizer followed by a 3 min ultrasound treatment in a Cole-Parmer 130-Watt Ultrasonic Processor at 25 °C.

### 2.6. ζ Potential, Particle Size, and Size Distribution of the Pickering Emulsions Droplets

The droplet size distribution, polydispersity index, and *ζ* potential of the emulsions (stabilized with NP1 and NP2 at concentrations of 0 to 20%) were measured using a Malvern Nano-S90 laser particle size analyzer 8 h after preparation at 25 °C [7].

### 2.7. Emulsion Stability

The stability of the emulsions with time was measured using a Turbiscan Lab Expert (Formulaction, Toulouse, France). The measurement principle of this instrument has been explained by Zequan et al. [31]. By scanning the sample at specific intervals, a pattern of the light flux (transmitted or backscattered) as a function of the sample height can be obtained, which is characteristic for each sample, so a parameter called Turbiscan Stability Index (TSI) can be calculated with the aid of the following equation:

(1) $$TSI=\sqrt{\frac{\sum_{i=1}^{n}{\left(x_{i}-x_{bs}\right)}^{2}}{n-1}}$$

where *x_i_* is the average backscatter per minute of measurement, *x_bs_* is the average value of *x_i_*, and *n* is the number of scans [6]. The TSI includes all the variations occurring in the system including the most common instability mechanisms that occur in food emulsions (creaming, flocculation, sedimentation, coalescence, Ostwald ripening, and phase inversion) [32]. The higher the TSI value, the lower the stability of the emulsion. A sample (25 mL) of the Pickering emulsion was added to the equipment glass cell and allowed to remain for 8 h at 25 °C and the Global TSI was then calculated as the arithmetic mean of the individual TSI values.

### 2.8. Emulsification Efficiency

The emulsification efficiency (EE%) is defined by the concentration of the incorporated material (squalene oil) in the Pickering emulsion over the initial concentration used to make the formulation. The EE % was calculated using Equation (2):

(2) $$EE\%=\frac{ml\;of\;encapsulated\;oil}{ml\;of\;total\;oil}\times 100$$

The amount of encapsulated oil was calculated as the amount of total oil minus the amount of free oil which was released from the system by centrifugation (3000 rpm for 10 min) immediately after the Pickering emulsion was formed [33].

### 2.9. Confocal Laser Scanning Microscopy (CLSM)

A Confocal Laser Scanning Microscope (CLSM) (LSM 710 NL0 Carl Zeiss, Oberkochen, Germany) was employed to visualize the structure of the Pickering emulsion droplets 8 h after their preparation. Fluorescein isothiocyanate (FITC) was used to stain the α-LA nanoparticles [34], and Nile Red dye was used to stain the oil phase [6]. The freshly prepared Pickering emulsions (1 μL) were placed along with the dyes between two cover glasses in the CLSM and operated in fluorescence mode with an Apochromat-Plan objective (63×/1.4 Oil DIC M27) at 60×/1.4.

### 2.10. Statistical Analysis

The results were reported as the arithmetic mean ± standard deviation of three replicates. The Student t-test and one-way ANOVA were used to detect statistical differences among treatments (α = 0.05).

## 3. Results and Discussion

### 3.1. Preparation and Characterization of α-LA Nanoparticles

Dynamic light scattering is a powerful technique used to determine the size, size distribution, and surface charge (ζ potential) of nanoparticles, obtaining information about their stability and surface interaction with other molecules [35]. Table 1 shows the experimental values of these parameters for the α-LA nanoparticles prepared by the desolvation and cross-linking method from protein solutions with initial pH values of 9 (NP1) and 11 (NP2).

It can be noted that the nanoparticles prepared at pH 11 (NP2) were significantly larger (*p*≤ 0.05) than the nanoparticles prepared at pH 9 (NP1). The PDI value of the NP1 was significantly larger than the PDI of the NP2. PDI is a representation of the size distribution within certain particle populations. Values of PDI ≤ 0.02 are considered adequate since the nanoparticle distributions can be considered monodisperse [36]. The size and size distribution of the α-LA nanoparticles fall in the range of the results obtained by other authors using the desolvation method for the same protein [20,25,26,37]. The ζ potential for both α-LA nanoparticles was near −30 mV (−29.7 and −25.4 mV), which indicated that the two α-LA nanoparticle systems had good stability [26]. In the case of α-LA nanoparticles prepared by desolvation and cross-linking, a negative surface electric charge at neutral and basic values of pH has been reported [38]. A minimal ζ potential of −30 mV is required for electrostatically stable nanoparticles and a minimal of −20 mV for steric stabilization [39]. Our data are consistent with the ζ potential values observed for other albumin nanoparticles [26,37]. These results indicate that, at the pH values used in the preparation (9 and 11) of the α-LA nanoparticles, electrostatic repulsion may play an important role in the Pickering stabilization of emulsions.

**Figure 1:**
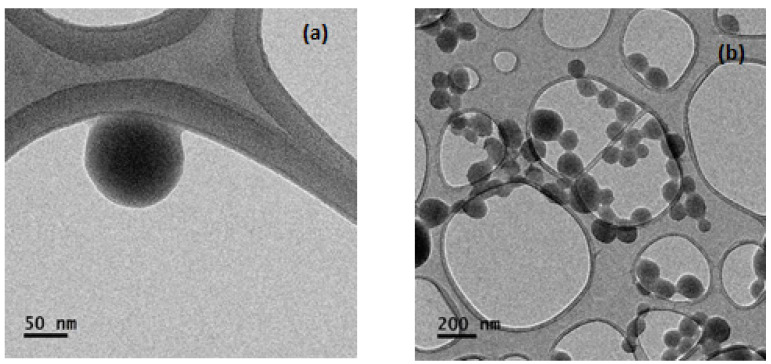
TEM images of α-LA nanoparticles formed from protein solutions with different pH values: (**a**) pH 7 and (**b**) pH 9.

It can be seen (Figure 1) that the morphology of the α-LA nanoparticles, as observed by SEM, is basically spherical with particle sizes of approximately 150 nm. These sizes are consistent with the sizes determined by DLS in this study and by other authors [25,37,38] by TEM.

**Figure 2:**
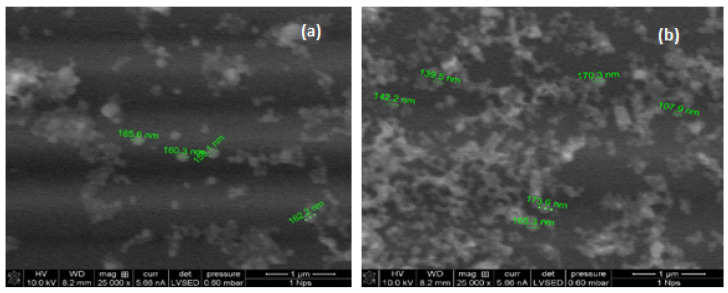
SEM images of α-LA nanoparticles formed from protein solutions with different pH values: (**a**) pH 7 and (**b**) pH 9.

SEM images were taken to evaluate the general and surface morphology of the nanoparticles. The nanoparticle size on the images matched, again, well the DLS data. Most of the α-LA nanoparticles have regular spherical shapes with average diameters of 150 nm (Figure 2) and are similar to the images of other SEM studies [26].

**Figure 3:**
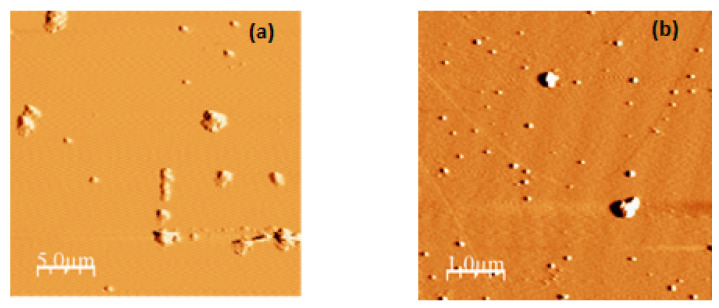
AFM images of α-LA nanoparticles formed from protein solutions with different pH values: (**a**) pH 7 and (**b**) pH 9.

Atomic force microscopy (AFM) has been effectively applied in the field of food proteins. There are many applications (nanoimaging, force spectroscopy, manipulation, etc.) of AFM; however, nanoimaging is the most popular method to evaluate the structures, functions, sizes, and distributions in proteins [30,40,41]. In the case of both kinds of α-LA nanoparticles, semispherical surfaces can be observed (Figure 3) with particle sizes in the range of 150 to 165 nm. Nanoparticle agglomerates can be distinguished in all cases. The nanoparticle size and size distribution were, again, very similar to the data obtained by DLS. This similarity was previously observed by Hoo et al. [41], who also indicated that AFM has advantages over DLS just in the case of non-monodisperse solutions. This is the first time that native α-LA nanoparticles have been visualized by AFM. The only antecedents were the observations of disk-shaped nanoparticles and nanotubes obtained upon partial enzymatic hydrolysis of α-LA [22,42].

### 3.2. Droplet Size, Polydispersity Index, and ζ Potential of the Pickering Emulsions

There are two main factors affecting the emulsion droplet size and size distribution during the preparation of Pickering emulsions. One of them is the concentration of α-LA nanoparticles initially dispersed in the aqueous phase and the other is the amount of amaranth oil phase. In this case, the second factor was maintained constant at 30% following the recommendation of Castañeda-Leal et al. [6]. The effect of the concentration of α-LA nanoparticles in the aqueous phase was observed by measuring the droplet size, polydispersity index, and ζ potential over a wide range of nanoparticle concentrations. The results can be observed in Table 2.

The Table shows that the average emulsion droplet size decreased from 5.8 ± 0.4 to 1.1 ± 0.5 μm and from 5.9 ± 0.2 to 2.2 ± 1.4 μm as NP1 and NP2 concentrations increased from 3 to 20% at a constant oil fraction (0.3). No significant difference between the two sets of droplet sizes (NP1 and NP2) could be detected according to the Student t-test for paired measurements (*p* > 0.05). This behavior has been previously described in the case of Pickering emulsions stabilized with soy protein isolate-chitosan [4] and starch [6] nanoparticles. This dependence of the droplet size on the concentration of stabilizing particles has been previously described and explained as a phenomenon in which larger amounts of solid particles are able to generate smaller droplets with limited coalescence due to the formation of an adsorbed layer of solid particles forming a stiff coating around the liquid droplets [43]. In the case of this study, the variation of the droplet size with the concentration of α-LA nanoparticles could be modeled with polynomial equations as follows:

D_NP1_ = −0.0028C^3^ + 0.088C^2^ − 0.9893C + 8.0672 (3)

D_NP2_ = 0.0122C^2^ − 0.4982C + 7.2848 (4)

where D_NP1_ and D_NP2_ are the diameters of the droplets of the emulsions stabilized with NP1 and NP2 and C is the concentration (%) of NP1 and NP2 in equations 3 and 4, respectively. The determination coefficients (R^2^) were 0.9998 and 1.0000, respectively. The average droplet size in this study was smaller than the sizes reported in other studies where the authors used other kinds of nanoparticles [4,6,8]. No significant difference in the droplet size was detected (*p* ≤ 0.05) as a result of the pH values used in the preparation (9 and 11) of the α-LA nanoparticles (NP1 and NP2).

Statistically, it is difficult to work with small samples which come from populations with large standard deviations since, in these cases, the false positive rate is considerable when evaluating significant differences using One-way ANOVA [44]. However, adequate samples for the statistical evaluation of significant differences in PDI could be obtained in the cases of NP1-10, NP1-15, and NP1-20. In this case, the smallest PDI values (0.42 and 0.49) were found when 10 and 15% of NP1 initially dispersed in the aqueous phase were used (see Table 2). PDI is one of the important parameters to study the stability of emulsions. It is known that PDI values below 0.5 are indicative of the reliability of the measurements and the uniform distribution of the droplets [45]. In the case of this study, the smallest PDI values (0.42 and 0.60) were obtained for the Pickering emulsions prepared with 10% NP1 and NP2, respectively. These values are similar to those obtained in other works [46].

ζ potential is a critical parameter for assessing the stability of Pickering emulsions and for choosing adequate wall materials to be used as stabilizers. Generally, emulsion stability has been associated with ζ potential values of ± 30 mV [7]. In the case of this study, values of ζ potential between −39.7 and −59.8 mV and between −33.4 and −48.7 mV were obtained for the emulsions prepared with NP1 and NP2, respectively (see Table 2). All the values fall in the range of values reported by other authors [7,47] and in the interval of stability for emulsions (± 30 mV).

### 3.3. Confocal Laser Scanning Microscopy (CLSM)

**Figure 4:**
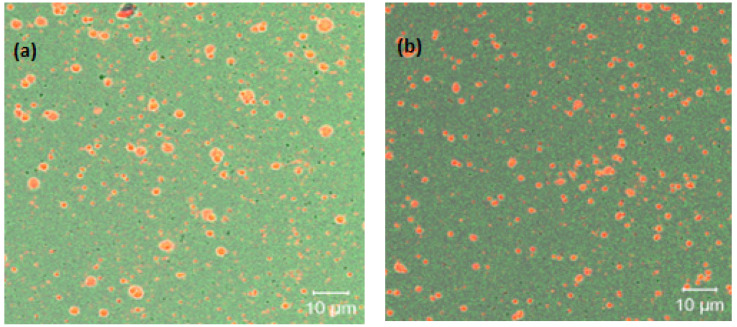
CLSM micrographs of amaranth oil Pickering emulsions stabilized with 15% NP1 (**a**) and NP2 (**b**).

In Figure 4a,b, images of the Pickering emulsions prepared with a 15% concentration of NP1 and NP2 are shown: red regions correspond to the oil phase and are surrounded by a white to green membrane (α-LA nanoparticles) on a light green background. Average droplet sizes between 3 and 4 μm can be observed along with some smaller droplets and individual agglomerates of nanoparticles stained in dark green. The thickness of the adsorbed layer of solid particles is between 700 and 1000 nm and depends on the size of the droplets to which they are adsorbed.

### 3.4. Stability of Pickering Emulsions Determined by Light Backscattering

**Figure 5:**
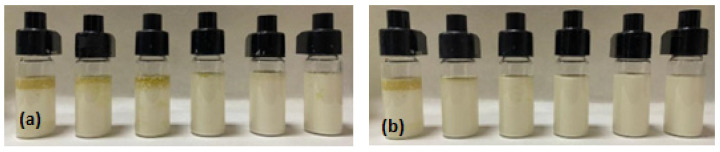
Photographs of the amaranth oil Pickering emulsions prepared with different concentrations (0, 3, 5, 10, 15, and 20%) of (**a**) NP1 and (**b**) NP2 after 8 h of storage at 25 °C.

Emulsion stability can be defined as “the ability of an emulsion to resist changes in its physicochemical properties over time” [32]. The stability of the emulsions with time was measured as the Global Turbiscan Stability Index (TSI). The Global TSI after 8 h of storage at different concentrations of NP1 and NP2 is shown in Table 2. The photographs of the Pickering emulsions after 8 h of storage are shown in Figure 5.

The Global TSI decreased from 8.63 to 1.98 as NP1 concentration increased from 3 to 15%, indicating an increase in the stability of the Pickering emulsions. No oil separation can be observed for the emulsions with 10, 15, and 20% of NP1 (Figure 5a). This was to be expected since they have the lowest values of Global TSI (Table 2). In the case of the emulsions stabilized with NP2, the Global TSI decreased from 4.7 to 1.7 as the nanoparticle concentrations increased from 3 to 20%. The oil separation could not be observed in the samples with 5, 10, 15, and 20% of nanoparticles (Figure 5b). Again, these concentrations formed the emulsions with the lowest Global TSI values (Table 2). This indicates that 10 and 5% are the concentrations necessary for NP1 and NP2 to reach the critical coverage ratio. If this ratio is not achieved, coalescence is likely to occur. There are many instability mechanisms occurring in food emulsions, however, under certain conditions, Ostwald ripening is considered the main mechanism in the case of Pickering emulsions [48]. However, the mechanism is inversely proportional to the droplet size. For droplets larger than 1 μm, the process occurs only at a very slow rate, which is probably the case of the system in this study. Global TSI values of 1.86 for NP1-10% and 1.6 for NP2-10% are the amaranth oil Pickering emulsions with the highest stabilities with time. These values are comparable to those obtained by Leal–Castañeda et al. [6] for Pickering emulsions stabilized with amaranth grain starch after 6 h of storage. The emulsified region in all the studied systems was located on the right side of the Turbiscan graphs, indicating that the instability region is located at the top of the emulsion and confirming that the main instability mechanism is creaming. Previous reports indicate that in the case of Pickering emulsions, the storage stability can be measured by creaming since these systems show high stability against coalescence [49].

### 3.5. Emulsification Efficiency

The higher emulsification efficiencies were obtained in the cases of the NP1-10% and NP2-10% emulsions with 92 and 94%, respectively (see Table 2). There are reports that in the case of polysaccharides, such as chitosan, the ζ potential is a good indicator of the emulsification efficiency of colloidal particles [49]. In the case of this study, the α-LA nanoparticles have ζ potential values between −29.7 and −25.4 mV, which apparently could indicate that a good emulsification efficiency could be achieved once the critical coverage ratio is reached.

Considering the characteristics of the Pickering emulsions prepared with different concentrations of NP1 and NP2 as stabilizers, the formulations with the best functionality were those containing NP1-10% and NP2-10% (see Table 2). These formulations have a good droplet size (4.15 and 3.52 μm), they are relatively homogeneous (PDI values of 0.42 and 0.60), they have a ζ potential indicative of high stability (−44.7 and −41.9 mV) which was corroborated by the lowest values of Global TSI (1.86 and 1.60), and they have the highest values of emulsification efficiency (92 and 98%). These formulations are, therefore, the best choices for the preparation of amaranth oil Pickering emulsions for the adequate delivery of squalene. Actually, they could have wide application possibilities as food delivery systems for lipophilic bioactive compounds. A previous study considered the possibility of preparing nanoparticle-stabilized Pickering emulsions to transform liquid oils into soft solids that could be used as a trans-fat-free replacement for partially hydrogenated oils utilized in margarines, shortenings, and bakery products [50].

## 4. Conclusions

Food grade squalene-rich amaranth oil Pickering emulsions stabilized by α-LA nanoparticles were successfully prepared. It was found that α-LA nanoparticles prepared by the desolvation and cross-linking method from protein solutions with pH values of 9 (NP1) and 11 (NP2) acted as good stabilizers for Pickering emulsions. Dependence of the droplet size of the emulsions on the nanoparticle concentration was observed. The critical coverage ratio was reached when 10 and 5% nanoparticle concentrations were used for NP1 and NP2, respectively. Our findings suggest that the NP1-10% and NP2-10% can be used as novel stabilizers for Pickering emulsions to provide protection for valuable lipophilic bioactive compounds such as squalene. This is the first time that native α-LA nanoparticles have been used as stabilizers of Pickering emulsions.

## Figures and Tables

**Table 1:** Effect of pH of the initial protein solution on the α-LA nanoparticles size, size distribution, and ζ potential.

Nanoparticle	Size (nm)	Polydispersity Index (PDI)	ζ Potential (mV)
NP1 (pH 9)	143 ± 8 ^a^	0.09 ± 0.01 ^a^	−29.7 ± 1.42 ^a^
NP2 (pH 11)	152 ± 1 ^b^	0.06 ± 0.04 ^b^	−25.4 ± 2.35 ^b^

Values in a column followed by different lowercase superscript letters were significantly different (α = 0.05) from each other according to the Student *t*-test for paired measurements. Data are expressed as the mean of 36 replicates ± standard deviation.

**Table 2:** Droplet size, polydispersity index, ζ potential, global turbiscan stability index, and encapsulation efficiency of emulsions at different concentrations of α-LA nanoparticles.

Concentration of α-LA Nanoparticles (%)	Droplet Size (μm)	PDI	ζ Potential	Global TSI	Emulsification Efficiency (%)
NP1-3	5.8 ± 0.4	0.73 ± 0.16	−59.8 ± 8.3	8.63 ± 0.7	40
NP1-5	5.0 ± 0.4	0.65 ± 0.17	−39.7 ± 1.95 ^a^	6.46 ± 1.3	48
NP1-10	4.15 ± 0.75	0.42 ± 0.03 ^a^	−44.7 ± 3.2 ^a,b^	1.86 ± 0.07 ^b^	92
NP1-15	3.6 ± 0.66	0.49 ± 0.10 ^a^	−39.7 ± 15.8 ^a,b^	1.98 ± 0.2 ^b^	84
NP1-20	1.1 ± 0.5	0.74 ± 0.04 ^b^	−40.6 ± 5.4	2.06 ± 0.7	87
NP2-3	5.9 ± 0.2	0.88 ± 0.12	−42.5 ± 3.4 ^a,b^	4.7 ± 2.7	46
NP2-5	5.1 ± 0.37	0.61 ± 0.11	−41.7 ± 5.4 ^a,b^	2.0 ± 0.7	51
NP2-10	3.52 ± 0.34	0.60 ± 0.16	−41.9 ± 20	1.6 ± 0.04 ^a^	94
NP2-15	2.56 ± 0.36	0.66 ± 0.16	−33.4 ± 9.9	1.8 ± 0.8	86
NP2-20	2.2 ± 1.4	0.88 ± 0.15	−48.7 ± 3.7 ^b^	1.7 ± 0.1 ^a,b^	89

Different superscript lowercase letters within the same column indicate significant difference (α = 0.05). Only the samples with the smaller standard deviations were tested. Data are expressed as the mean of 3 replicates ± standard deviation. NP1 and NP2 are nanoparticles prepared at pH 9 and 11.

## Data Availability

The data presented in this study are available on request from the corresponding authors.
